# Baohuoside I Inhibits Osteoclastogenesis and Protects Against Ovariectomy-Induced Bone Loss

**DOI:** 10.3389/fphar.2022.874952

**Published:** 2022-04-27

**Authors:** Min Ma, Ao-yuan Fan, Zheng Liu, Li-qing Yang, Jun-ming Huang, Zhi-ying Pang, Feng Yin

**Affiliations:** ^1^ Department of Joint Surgery, Shanghai East Hospital, School of Medicine, Tongji University, Shanghai, China; ^2^ Department of Orthopaedic, the First Affiliated Hospital of Nanchang University, Nanchang, China; ^3^ Shanghai Institute of Stem Cell Research and Clinical Translation, Shanghai, China; ^4^ Shanghai Clinical Research Centre for Ageing and Medicine, Shanghai, China

**Keywords:** osteoclast, baohuoside I, uPAR, MAPK, NF-kB, RANKL, osteoporosis

## Abstract

Bone-resorbing osteoclasts are essential for skeletal remodelling, and the hyperactive formation and function of osteoclasts are common in bone metabolic diseases, especially postmenopausal osteoporosis. Therefore, regulating the osteoclast differentiation is a major therapeutic target in osteoporosis treatment. Icariin has shown potential osteoprotective effects. However, existing studies have reported limited bioavailability of icariin, and the material basis of icariin for anti-osteoporosis is attributed to its metabolites in the body. Here, we compared the effects of icariin and its metabolites (icariside I, baohuoside I, and icaritin) on osteoclastogenesis by high-content screening followed by TRAP staining and identified baohuoside I (BS) with an optimal effect. Then, we evaluated the effects of BS on osteoclast differentiation and bone resorptive activity in both *in vivo* and *in vitro* experiments. In an *in vitro* study, BS inhibited osteoclast formation and bone resorption function in a dose-dependent manner, and the elevated osteoclastic-related genes induced by RANKL, such as NFATc1, cathepsin K, RANK, and TRAP, were also attenuated following BS treatment. In an *in vivo* study, OVX-induced bone loss could be prevented by BS through interrupting the osteoclast formation and activity in mice. Furthermore, mechanistic investigation demonstrated that BS inhibited osteoclast differentiation by ameliorating the activation of the MAPK and NF-kB pathways and reducing the expression of uPAR. Our study demonstrated that baohuoside I could inhibit osteoclast differentiation and protect bone loss following ovariectomy.

## Introduction

Osteoporosis is a systemic metabolic disease characterized by a deteriorated bone microarchitecture, reduced bone mass, and impaired bone strength, resulting in an increased risk of fragility fracture (Compston et al., 2019). Osteoporotic fractures are a major source of morbidity and mortality in the ageing population and place significant medical and economic challenges on the society (Clynes et al., 2020; Rashki Kemmak et al., 2020). Because the pathophysiology of osteoporosis is the uncoupling of osteoclasts and osteoblasts, finding novel agents inhibiting bone resorption or stimulating new bone formation is considered a feasible preventive and therapeutic strategy for osteoporosis (Khosla and Hofbauer, 2017; Wang et al., 2017; Gennari et al., 2020).

Over the past three decades, a variety of drugs, most of which are osteoclast depressants (anti-catabolic agents), such as oestrogens, raloxifene, and bisphosphonates, have been used for osteoporosis treatment. The side effects that occur during the treatment may cause other problems (Khosla and Hofbauer, 2017; Li et al., 2021). Bisphosphonates are the first-line drugs used to reduce bone mass loss, but their long-term usage may lead to severe adverse effects, namely, atypical femoral fractures and osteonecrosis of the jaw (Papapetrou, 2009; Meyyur Aravamudan and Er, 2019). Oestrogen-based hormone replacement therapy is only recommended for postmenopausal women, and the selective oestrogen receptor modulator raloxifene may be associated with an increased incidence of blood clots and strokes (Barrett-Connor et al., 2005). Denosumab or romosozumab are emerging drugs for the treatment of osteoporosis with excellent therapeutic effects, but the reported side effects are also worth considering, namely, musculoskeletal pain, hypercholesterolaemia, serious infection, skin reaction, and arterial calcification (Fuggle et al., 2020). Given the growing global osteoporosis population, it is necessary to introduce a safer and more effective drug for osteoporosis.

Osteoclasts are multinucleated cells responsible for bone resorption that originate from haematopoietic monocytes or macrophages (Bar-Shavit, 2007). Apart from differentiation, cell–cell fusion is also an important step in osteoclast maturation. Being controlled mainly by M-CSF and RANKL, the monocytic precursors can fuse into osteoclasts (Dufrançais et al., 2021). During osteoclast differentiation, the macrophage colony-stimulating factor (M-CSF or CSF-1) and receptor activator of NF-kB ligand (RANKL) are deemed the two most important cytokines for osteoclast maturation and survival (Boyle et al., 2003). M-CSF was considered the first essential factor to maintain bone marrow monocyte (BMM) and preosteoclast vitality and promote osteoclast precursor differentiation and maturation through binding to receptor of the colony-stimulating factor-1 (CSF-1R) (Hamilton, 1997). The other essential cytokine, RANKL, participates in osteoclast differentiation by interacting with its receptor RANK (Park et al., 2017). The binding complexes of RANKL and RANK initiate the recruitment of tumour necrosis factor receptor–associated factors (TRAFs), then activate the nuclear factor-kB (NF-kB) and mitogen-activated protein kinase (MAPK) pathways and eventually result in enhanced transcription of Fos Proto-Oncogene (c-Fos) and nuclear factor of the activated T-cell cytoplasmic 1 (NFATc1) (Lomaga et al., 1999; Yuan et al., 2015; Seo et al., 2020). In addition to CSF-1R and RANK, the urokinase-type plasminogen activator receptor (uPAR) also plays a crucial role in governing osteoclastogenesis (Huang et al., 2018a). According to a study by Kalbasi Anaraki et al. (2015), the elevation of uPAR potentiates the RANKL-induced osteoclast differentiation of BMMs through the PI3K/Akt signalling pathway. Intriguingly, Kanno et al. (2016) demonstrated that blocking uPAR suppressed lipopolysaccharide-induced inflammatory osteoclastogenesis. Based on these studies, uPAR plays a crucial role in maintaining bone homeostasis. Small molecular compounds regulating uPAR could be effective alternative therapeutics for osteoporosis treatment.

Traditional Chinese herbs have been used in medical practice for thousands of years in East Asian countries. Herba Epimedii, the dried leaves of the medicinal plant Epimedii, has been commonly considered a tonic and aphrodisiac, as well as an antirheumatic and anti-osteoporotic agent in China (Zhang et al., 2009). Flavonoids are the major effective ingredient of Herba Epimedii, and icariin is the most abundant component in Epimedium flavonoids; therefore, icariin is used as a standard for the quality control of Epimedii (Wu Y. et al., 2017). It has been reported that icariin exerts a wide range of biological activities, such as antioxidant, immunomodulatory, cardioprotective, and neuroprotective activities (Chen et al., 2017; Wu et al., 2018; Wang et al., 2020; Zhou et al., 2020). In the skeletal system, icariin has been demonstrated to have a positive effect on bone metabolism, and treatment with icariin has resulted in suppressed osteoclast differentiation, improved commitment differentiation of the mesenchymal stem cells, and improved maturation and mineralization of osteoblasts after oestrogen withdrawal (Huang et al., 2017; Sun et al., 2020). However, it has been confirmed that the bioavailability of oral icariin is limited in rats (Xu et al., 2007). Icariin can be transformed by human intestinal microflora, and the biotransformed derivatives of icariin, such as baohuoside I, icariside I, and icaritin, exhibit not only excellent absorbability but also more potent pharmacological effects than icariin (Chen et al., 2011). Previous studies have concentrated on the pharmacological activities of icariin, but there have been few reports on the effects of icariin metabolites on osteoporosis. In this study, we aimed to identify which of the main derivatives of icariin show optimal suppression of osteoclastogenesis and to identify potential mechanisms.

## Materials and Methods

### Reagents

Recombinant RANKL and M-CSF were purchased from PeproTech (Princeton, NJ, United States). Icariside I (ICS; C_27_H_30_O_11_; MW: 530.53), baohuoside Ⅰ (BS; C_27_H_30_O_10_; MW: 512.52), icariin (ICA; C_33_H_40_O_15_; MW: 676.68), icaritin (ICT; C_21_H_20_O_6_; MW: 368.38), and a Cell Counting Kit-8 (CCK-8) were purchased from Target Molecule Corp. (Boston, MA, United States). Trypsin-EDTA (0.05%), PBS, FBS, and MEM-Alpha basic were purchased from Gibco. Antibodies against p38 (#8690), p-p38 (#4511), ERK (#9102), p-ERK (#4370), JNK (#9252), p-JNK (#4668), p65 (#8242), p-p65 (#3033), IκBα (#4812), p-IκBα (#2859), and RANK (#4845) were purchased from Cell Signaling Technology (Boston, MA, United States). Antibodies against TRAP (ab52750) and cathepsin K (ab19027) were purchased from Abcam (Cambridge, MA, United States). Antibodies against NFATc1 (66963-1-Ig), uPAR (10286-1-AP), and β-actin (66009-1-Ig) were purchased from Proteintech Group Inc. (Wuhan, China). DAPI and Actin-Tracker Green were obtained from Beyotime (Shanghai, China). Cy3-labelled goat anti-rabbit antibody was purchased from Boster (Wuhan, China). Unless noted otherwise, other reagents were of the highest purity available and were obtained from Sigma-Aldrich (St. Louis, MO, United States).

### Cell Culture

As described previously, the bone marrow suspension was isolated from the tibia and femur bone marrow cavities of 4- to 6-week-old mice by α-MEM medium flushing. Twenty-four hours later, the nonattached bone marrow monocytes (BMMs) were collected and used for further experiments (Huang et al., 2018b). In all experiments, M-CSF (30 ng/ml) was used to maintain the survival of BMMs.

### Cell Counting Kit-8 Assay

The cytotoxic effects of ICS, BS, ICA, and ICT on the BMMs were evaluated by the CCK-8 assay. The BMMs were seeded at a density of 5 × 10^3^ cells/well in 96-well plates. Then, different concentrations of ICS, BS, ICA, and ICT (0.01, 0.1, and 1 μM) were applied every 2 days for 7 days. The CCK-8 reagent was used to measure the cell vitality every other day by recording the absorbance at a wavelength of 450 nm on a FlexStation 3 (Molecular Device, Shanghai).

### High-Content Screening

To quantify F-actin formation during osteoclast maturation, automated cellular imaging was performed by using the CellInsight CX7 High Content Analysis Platform (Thermo Fisher Scientific, United States). BMMs of 1 × 10^4^ were cultured and induced in 96-well black clear-bottom plates (Corning, United States). After osteoclasts formed, the cells were fixed (4% PFA, 20 min) and permeabilized (0.25% Triton X-100, 5 min). Then, F-actin staining was performed with Actin-Tracker Green (1 h), and eventually DAPI was employed for nuclear staining (5 min). Washes were performed three times with PBS. A total of 25 imaging fields per well were obtained with a ×10 objective with 2 × 2 binning. The nuclei were identified by DAPI-positive staining under a 488-nm widefield channel, while the cell skeleton elements and F-actin formation were identified by Actin-Tracker Green under a 565-nm widefield channel. The BMMs were identified as valid objects if they had DAPI-positive nuclei, and segmentation was performed by the shape method. Quantification of F-actin was performed by either a Circ Mask or Ring Mask with the adjustment of three positive pixels, representing the nuclei and cytoplasm, respectively. All the assays evaluating the inhibitory effects of ICA and its metabolites (ICS, BS, and ICT) under different concentrations (0, 0.01, 0.1, and 1 µM) were run with three replicates per condition and repeated with at least two biological replicates.

### In Vitro Osteoclast Differentiation Assay

The BMMs (1 × 10^4^) were incubated with RANKL and different concentrations of ICS, BS, ICA, and ICT (0.01, 0.1, and 1 μM). The cell culture medium was replaced every day until mature osteoclasts were formed (Wu et al., 2015). TRAP Staining was performed after fixation. Visual images of osteoclasts were acquired by using an Olympus IX-71 microscope (OLYMPUS, Japan). TRAP Stain–positive multinucleated cells with three or more nuclei were considered mature osteoclasts.

### Pit Formation Assays

The BMMs (2 × 10^4^) were seeded onto Corning Osteo Assay Surface plates (Corning Incorporated Life Science, NY, United States). The BMMs were stimulated with RANKL for 3 days. Then, different concentrations of BS (0.01, 0.1, and 1 μM) were supplemented until the osteoclasts were formed. All discs were washed with 5% sodium hypochlorite for 5 min followed by PBS for 10 min. Images were taken by light microscopy, and then the resorption pits were quantified by ImageJ.

### Immunofluorescence Staining of F-Actin and Urokinase-Type Plasminogen Activator Receptor

The BMMs (1 × 10^4^) were incubated with RANKL and different concentrations of BS (0.01, 0.1, and 1 μM). After observing the mature osteoclasts, the cells were fixed (4% PFA, 20 min), permeabilized (0.25% Triton X-100, 5 min), blocked (2% BSA, 1 h), and incubated with antibody against uPAR at 4°C overnight followed by Cy3-conjugated secondary antibody (1 h) at room temperature and then incubated with Actin-Tracker Green (1 h) to label F-actin and DAPI to label the nuclei (5 min). Fluorescent images were obtained by using a fluorescence microscope.

### RNA Extraction and Quantitative Reverse-Transcription Polymerase Chain Reaction

The BMMs (2.5 × 10^5^) were cultured with M-CSF, RANKL, and BS for 3 days. The total RNA was extracted by an RNA-Quick Purification Kit (YiShan Biotechnology, Shanghai), and an equal amount of RNA (1 μg) was mixed with the PrimeScript RT Master Mix kit (TaKaRa, Japan) to synthesize cDNA. Then, the synthesized cDNA was used to perform RT-qPCR with SYBR qPCR Mix (Yeasen, Shanghai) on a ABI QuantStudio5 (Q5) instrument. The relative expression levels of the target genes were calculated and normalized to β-actin. The primers used in the RT-qPCRs are presented in Table 1.

**TABLE 1:** List of primers used in quantitative real-time RT-PCR.

Target gene	Sense sequence (5′ to 3′)	Antisense sequence (5′ to 3′)
NFATc1	CAA​CGC​CCT​GAC​CAC​CGA​TAG	GGG​AAG​TCA​GAA​GTG​GGT​GGA
Rank	CAG​GAG​AGG​CAT​TAT​GAG​CA	GGT​ACT​TTC​CTG​GTT​CGC​AT
TRAP	TAC​CTG​TGT​GGA​CAT​GAC​C	CAG​ATC​CAT​AGT​GAA​ACC​GC
Cathepsin K	TGT​ATA​ACG​CCA​CGG​CAA​A	GGT​TCA​CAT​TAT​CAC​GGT​CAC​A
β-actin	GGC​TGT​ATT​CCC​CTC​CAT​CG	CCA​GTT​GGT​AAC​AAT​GCC​ATG​T

### Western Blot Analyses

The BMMs (2.5 × 10^5^) were pretreated with different concentrations of BS (0.01, 0.1, and 1 μM) in FBS-free medium for 3 h. After that, all cells were induced by RANKL for 1 h. To analyse the long-term action of BS on osteoclastogenesis, the BMMs were cultured with M-CSF, RANKL, and BS (1 μM) for 5 days. Protein was lysed by RIPA buffer (Beyotime, Shanghai, China) and quantified by a BCA assay kit (Thermo Fisher Scientific, MA, United States). Equal amounts of protein (10 μg) were subjected to 10% SDS polyacrylamide gel electrophoresis and transferred to PVDF membranes (Millipore, MA, United States). Protein Free Rapid Blocking Buffer (EpiZyme, Shanghai) was used to block the membranes followed by incubation with the respective antibodies overnight at 4°C and HRP-conjugated secondary antibodies for 1 h at room temperature. Subsequently, the membranes were immersed in Western ECL Substrate (Yeasen, Shanghai), and the protein bands were detected on a Tanon imaging system. Finally, analyses of greyscale images were obtained by using the ImageJ software.

### Ovariectomy Murine Model Establishment

Animal care and experimental procedures were approved by the Animal Use and Care Committee of Tongji University (Shanghai, China). 12-week-old female C57/BL6 mice were purchased from SLAC Laboratory Animal Co., Ltd. (Shanghai, China) and fed in the animal care facility of Tongji University. They were randomly distributed into three groups (n = 10 mice/group): the sham-operated mice receiving intraperitoneal equal volume DMSO injection (SHAM); the bilaterally ovariectomized mice receiving intraperitoneal DMSO injection (OVX); and the bilaterally ovariectomized mice receiving intraperitoneal BS injection (10 mg/kg) (BS). BS and DMSO were administered every other day for 6 weeks since starting on the third day after surgery. After 6 weeks of drug administration, all the mice were sacrificed for further investigation.

### Micro–Computed Tomography and Histomorphometric Analysis

Femoral specimens were scanned and analysed by a Scanco viva CT 40 instrument (Scanco Medical, Basserdorf, Switzerland) according to a previous study (Wu et al., 2017). Indices such as BV/TV, Tb.N, Tb.Th, and Tb. Sp were quantitatively measured, and 3D images were obtained from the sagittal, coronal, and transverse positions by using a built-in software. Femur specimens were decalcified at room temperature for 3 weeks. All the samples were then embedded in paraffin and sectioned into 5-µm-thick slices. Haematoxylin and eosin (H&E) staining was performed to evaluate the trabecular structure. TRAP staining was performed, and the number of osteoclasts near the femoral metaphysis was counted.

### Immunohistochemistry

Briefly, after dewaxing with xylene, the blank sections of femurs were heated in a citrate-EDTA buffer (100°C, 10 min), and the endogenous peroxidase was inactivated by 3% H_2_O_2_ (37°C, 15 min). Subsequently, the treated sections were blocked with goat serum (37°C, 1 h) and incubated with uPAR antibody (4°C, 18 h). The next day, a DAB Substrate Kit (Solarbio, Beijing, China) was used to evaluate the level of protein expression after secondary antibody treatment. After photographing, quantitative analysis of the positive immunohistochemical expression was performed by using ImageJ software.

### Serum Biochemistry

The serum levels of uPAR, β-CTX, CTX-I, PINP, OCN, ON, and OPN were evaluated by a mouse ELISA kit (Boster, Wuhan, China) according to the operation manual.

### Statistical Analysis

All the experiments were independently repeated more than three times, and all the quantitative results are shown as mean ± standard deviation (SD). The difference between the two groups was verified by *t* test, and one-way ANOVA was used to analyse more than two groups. *p* values less than 0.05 were considered statistically significant.

## Results

### Effects of Icariin and Its Metabolites (Icariside, Baohuoside Ⅰ, and Icaritin) on the Cell Viability of Bone Marrow Monocytes

**FIGURE 1:**
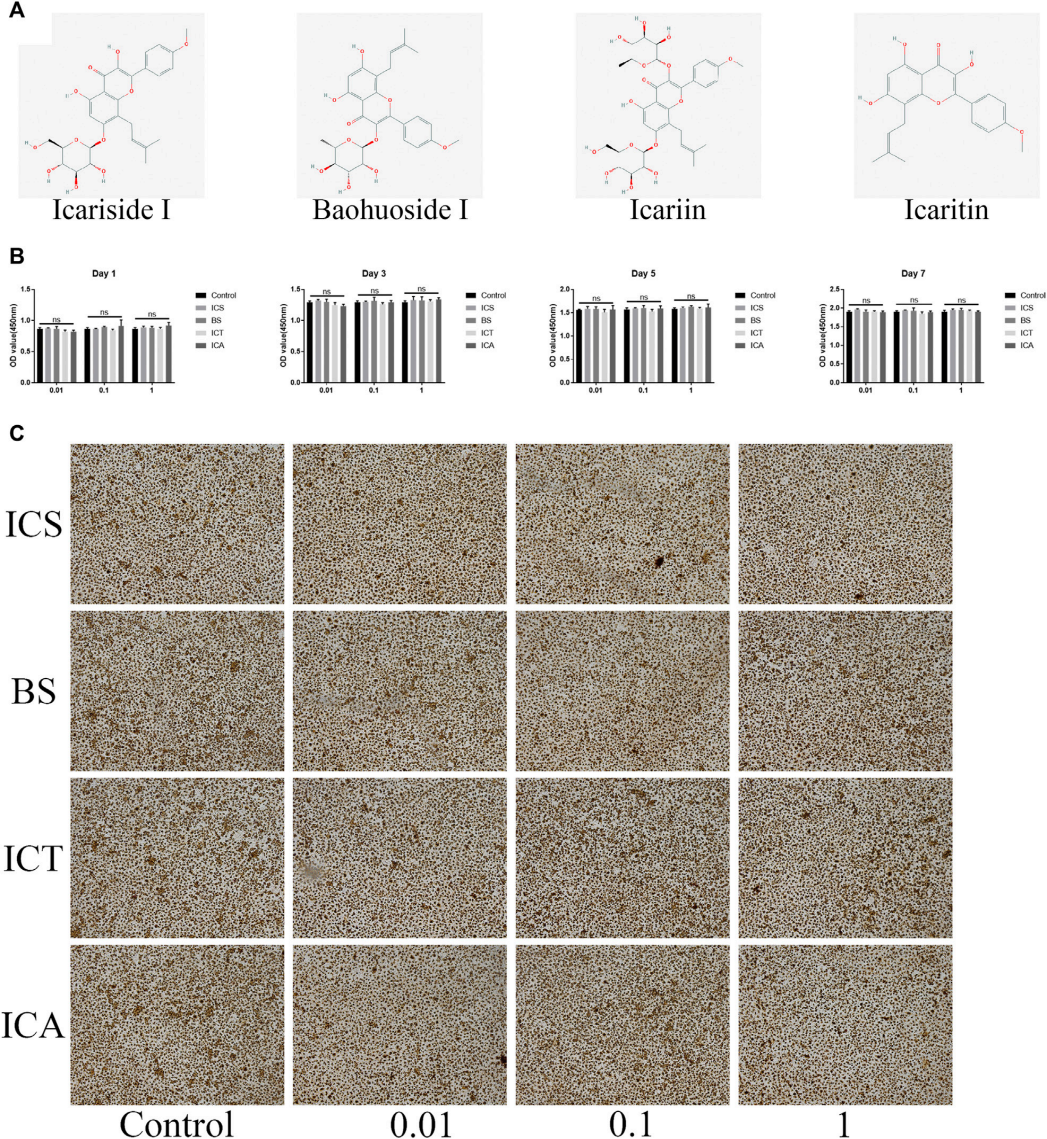
ICA and its metabolites (ICS, BS, and ICT) showed no toxic effect on the BMMs. **(A)** The structural formula of ICA and its metabolites. **(B)** CCK-8 assay was performed with different concentrations of ICS, BS, ICT, and ICA in the BMMs for 1, 3, 5, and 7 days and indicated that all the compounds have no toxic effect on BMMs. **(C)** TRAP Staining showing that different concentrations of ICS, BS, ICT, and ICA have an effect on the BMMs morphology for 7-days culture without RANKL stimulation. All bar graphs are presented as the mean ± SD; n = 3. ns, not statistically significant.

The structural formula of ICA and its metabolites are shown in Figure 1A. First, the cytotoxic effects of different concentrations of ICA and its metabolites (0, 0.01, 0.1, and 1 µM) on the BMMs were evaluated by the CCK8 assay. The results showed that the different concentrations of ICA and its metabolites had no cytotoxic effect on the BMMs over 7 days (Figure 1B). After 7 days of culture, we stained all the cells with TRAP and found that ICA and its metabolites had no effect on the morphology of the cells (Figure 1C).

### High-Content Screening of the Inhibition Effects of Icariin and Its Metabolites on F-Actin Formation

**FIGURE 2:**
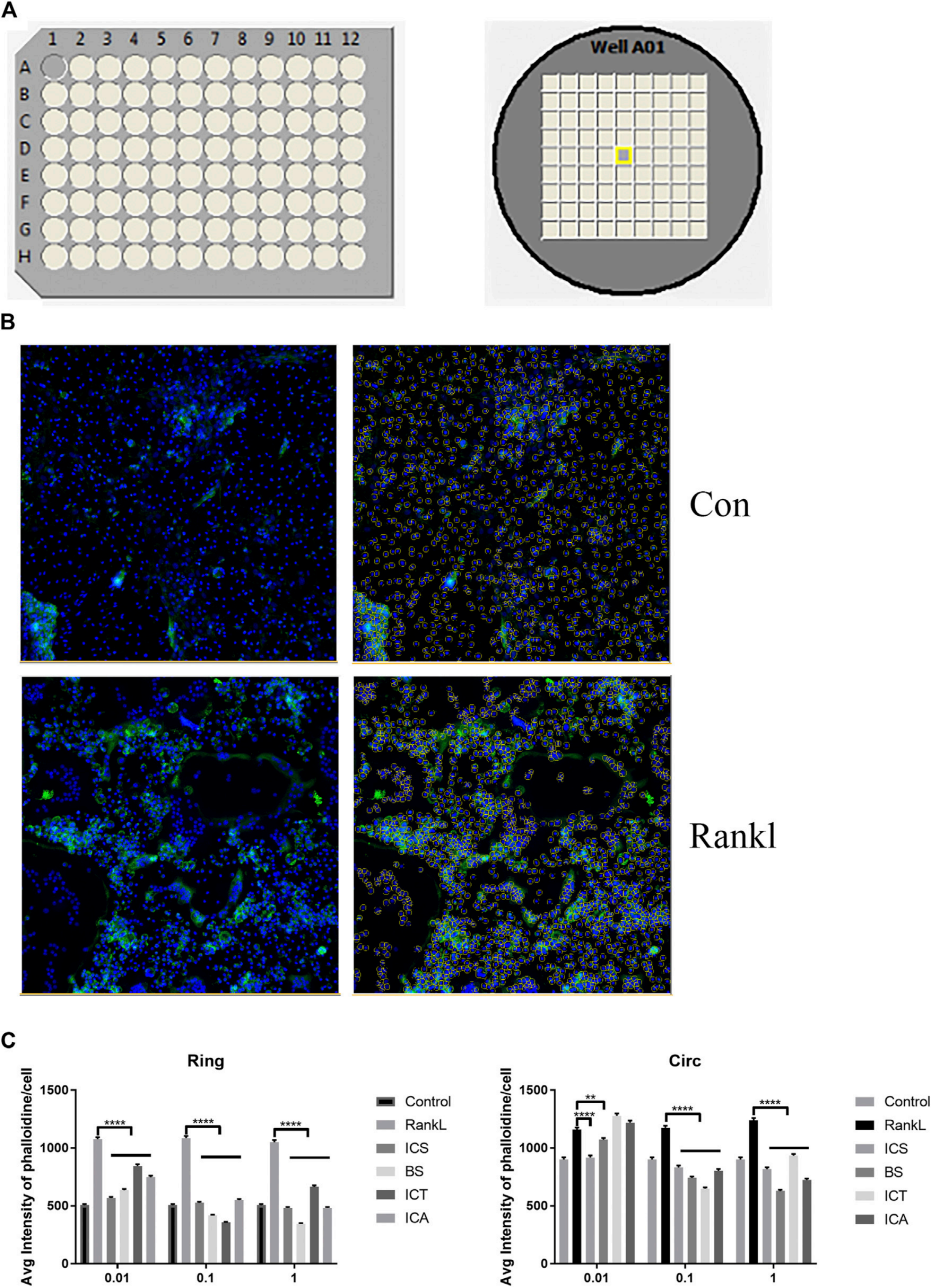
High-content screening showing BS best inhibits F-actin formation. **(A)** The scheme of high-content screening protocol. The BMM cells were seeded onto 96-well plates until osteoclasts formation. **(B)** Representative quantitative analysis image of auto-segmentation based on nuclei identification. **(C)** The inhibition effect of different concentrations of ICA and its metabolites (0, 0.01, 0.1, and 1 µM) against RANKL-induced F-actin formation on BMMs were evaluated by the general intensity measurement assay. All bar graphs are presented as mean ± SEM; n = 3. **p < 0.01, ****p < 0.0001.

The scheme of the high-content screening protocol is shown in Figure 2A. The inhibitory effects of the different concentrations of ICA and its metabolites (0, 0.01, 0.1, and 1 µM) against RANKL-induced F-actin formation on the BMMs were evaluated by a general intensity measurement assay. We found that ICA and its metabolites, namely, ICS, BS, and ICT (0, 0.01, 0.1, and 1 µM), inhibited F-actin formation. The quantitative analysis indicated that, compared with ICA and other metabolites, BS could best inhibit F-actin formation at three concentrations (Figures 2B,C).

### Icariin and Its Metabolites Inhibit Receptor Activator of Nuclear Factor-kB Ligand–Induced Osteoclast Differentiation

**FIGURE 3:**
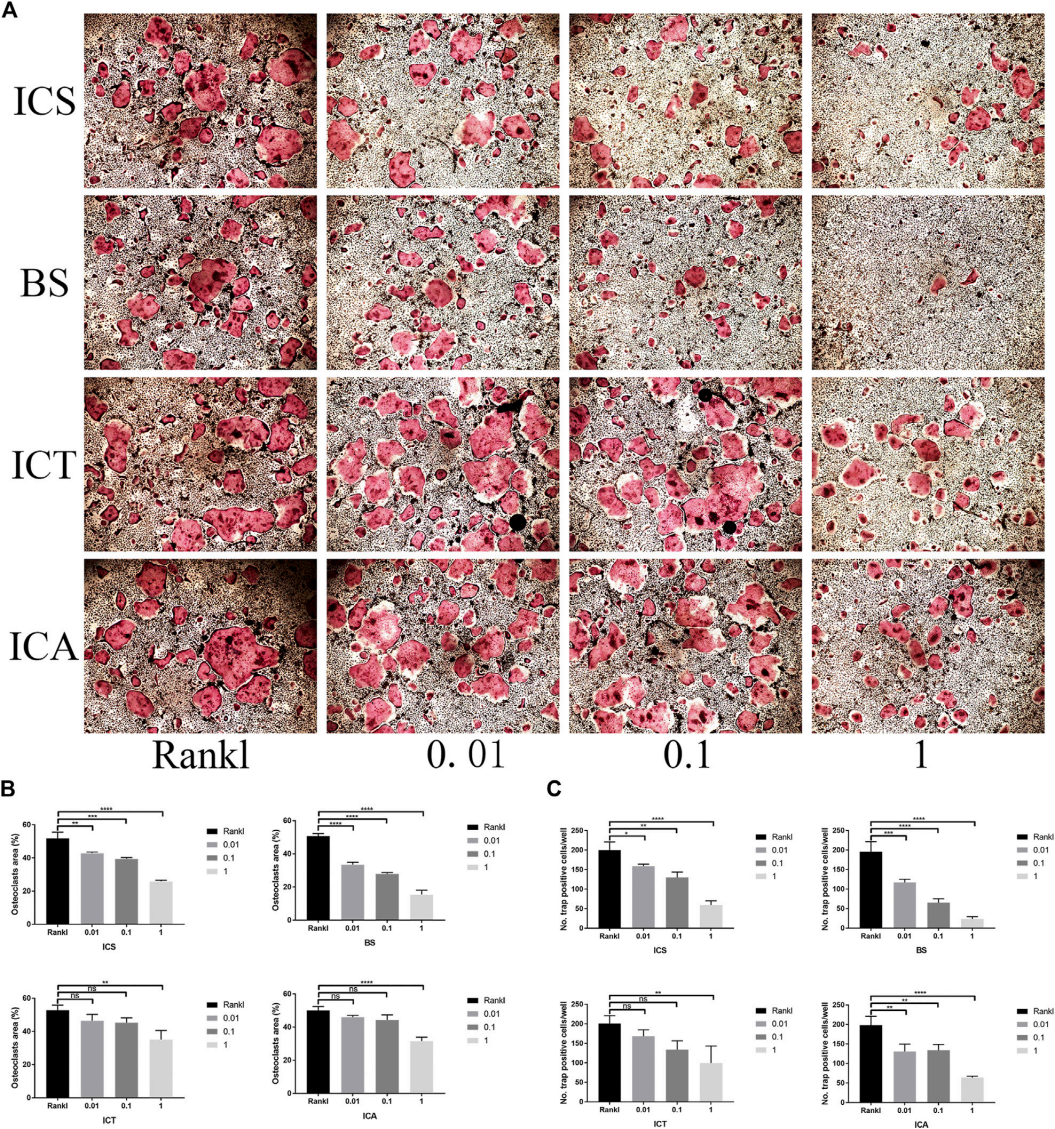
ICA and its metabolites (ICS, BS, and ICT) suppressed RANKL-induced osteoclastogenesis, and BS showed the most excellent effect. **(A)** TRAP Staining shows the different concentrations of ICS, BS, ICT, and ICA inhibiting osteoclast formation **(B,C)** TRAP-positive cells and area were quantified after the administration of different concentrations of ICS, BS, ICT, and ICA and indicated that BS showed optimal effect on suppressing osteoclast differentiation at different concentrations. All bar graphs are presented as mean ± SD; n = 3. ∗*p* < 0.05; ∗∗*p* < 0.01; ****p < 0.0001; ns, not statistically significant.

Various amounts of ICS, BS, ICT, and ICA (0, 0.01, 0.1, and 1 µM) were added to the osteoclast-inducing medium to assess the effects on osteoclast formation. According to the formation of osteoclasts, all the treatments inhibited osteoclast differentiation in a dose-dependent manner (Figure 3A), but different concentrations of each reagent demonstrated significant differences in osteoclast amounts and sizes (Figures 3B,C). Compared with ICA and other metabolites, BS showed the optimal effect on suppressing osteoclast differentiation at different concentrations, so BS was selected for follow-up experiments.

### Baohuoside Ⅰ Reduces Osteoclastic Bone Resorption

**FIGURE 4:**
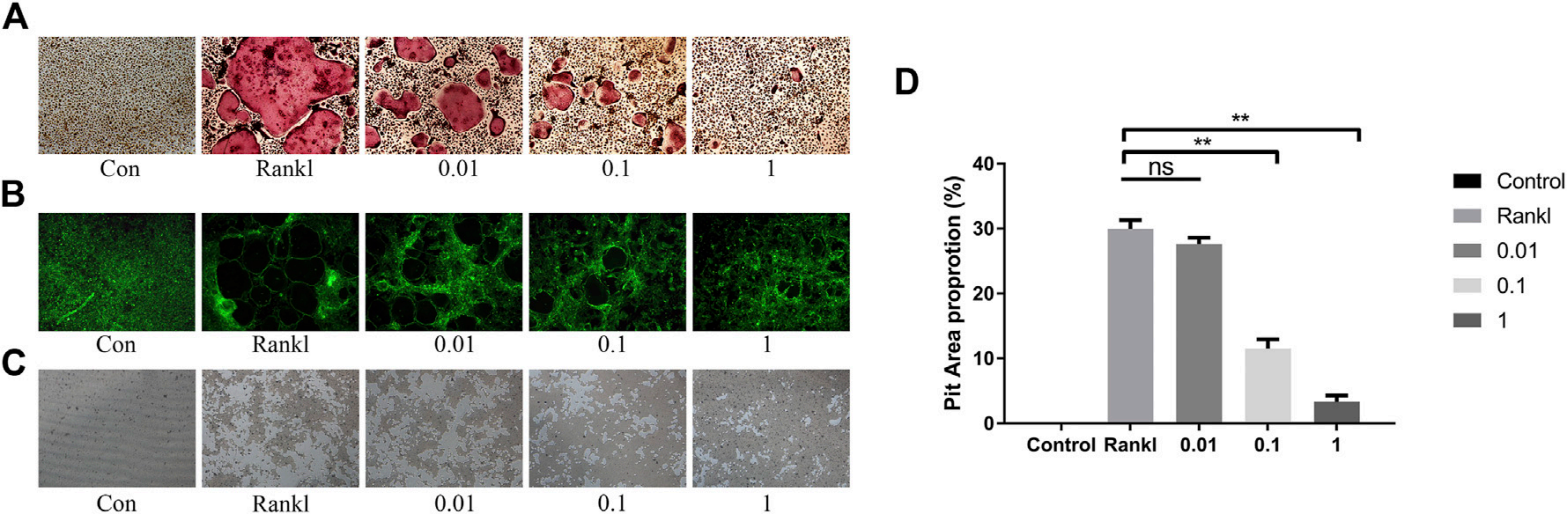
BS restrained F-actin formation and bone resorption. **(A)** Representative images of TRAP-stained osteoclasts on the Osseo Assay plate. **(B)** F-actin ring assessed by Actin-Tracker staining. **(C)** Representative images of pit formation by osteoclasts on the Osseo Assay plate. **(D)** Quantification of pits formation area. All bar graphs are presented as mean ± SD; n = 3. ∗∗*p* < 0.01; ns, not statistically significant.

Next, we assessed the effect of BS on osteoclast function by a pit formation assay. The BMMs were cultured and induced in Osseo Assay plates by BS. Both the number and size of the osteoclasts were attenuated as the concentration of BS increased (Figure 4A). In addition, the staining results indicated that BS treatment inhibited the formation of the F-actin ring, which is considered critically important in osteoclastic function (Figure 4B). Consistent with the inhibition of the F-actin ring, the bone resorption area was also reduced after BS treatment (Figures 4C,D).

### Baohuoside Ⅰ Inhibits Osteoclastogenesis by Suppressing the Activated Mitogen-Activated Protein Kinase and Nuclear Factor-κB Pathways

**FIGURE 5:**
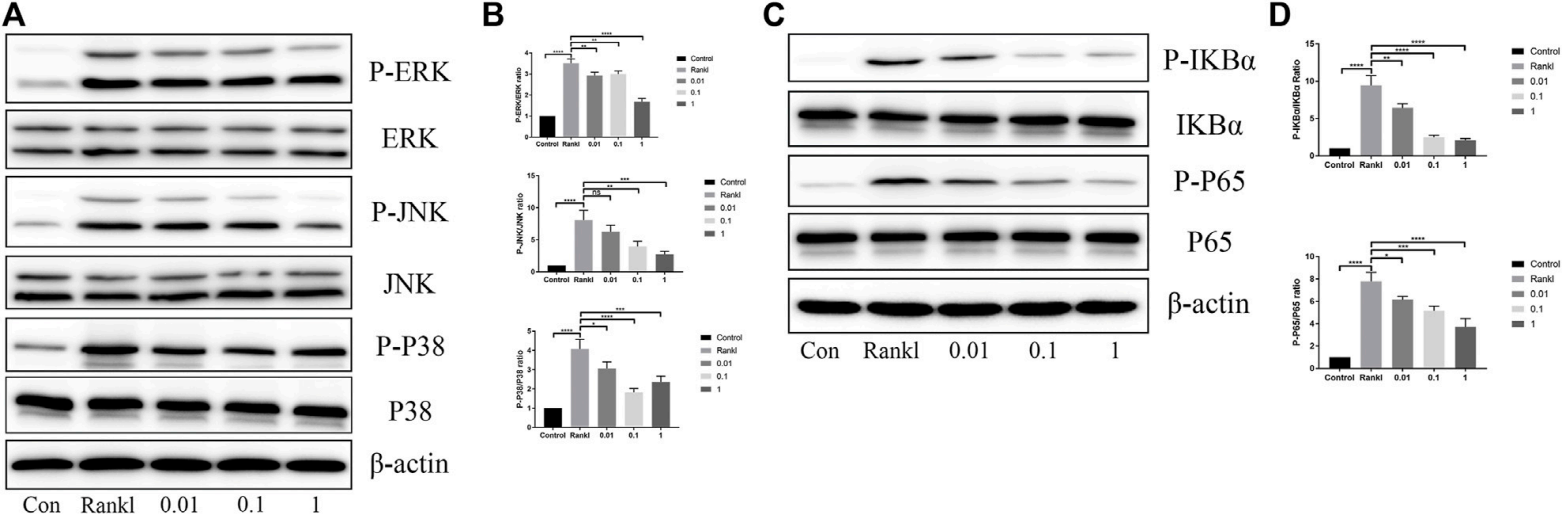
BS suppressed RANKL-induced activation of MAPKs and NF-kB. **(A)** Representative western blot of ERK, JNK, and p38 phosphorylation treated with BS after RANKL induction. **(B)** Quantitative analysis of phosphorylated ERK, JNK, and p38 to total ERK, JNK, and p38. **(C)** Representative western blot of p65 degradation and IkB-α phosphorylation treated with BS after RANKL induction. **(D)** Quantitative analysis of phosphorylated IkB-α and p65 to total IkB-α and p65. All bar graphs are presented as mean ± SD; n = 3. ∗*p* < 0.05; ∗∗*p* < 0.01; ∗∗∗*p* < 0.001; ****p < 0.0001.

There is no doubt that the activation of the MAPK and NF-κB pathways is indispensable during osteoclast differentiation; thus, we investigated the effects of BS on these two pathways. As shown in immunoblotting, the phosphorylated levels of ERK, P38, and JNK were reduced after the administration of BS. Moreover, the downregulation ratio was related to the concentration of BS, except for the lowest levels of phosphorylated P38, which was noted at a concentration of 0.1 µM (Figures 5A,B). Concerning the NF-κB pathway, the BS treatment inhibited the phosphorylation and degradation of IkBα, and subsequently, the increased phosphorylation of P65 induced by RANKL was also ameliorated (Figures 5C,D).

### Baohuoside Ⅰ Abrogates Receptor Activator of Nuclear Factor-kB Ligand–Associated Nuclear Factor of the Activated T-Cell Cytoplasmic 1 Activation and Downregulates Osteoclast-Related Genes

**FIGURE 6:**
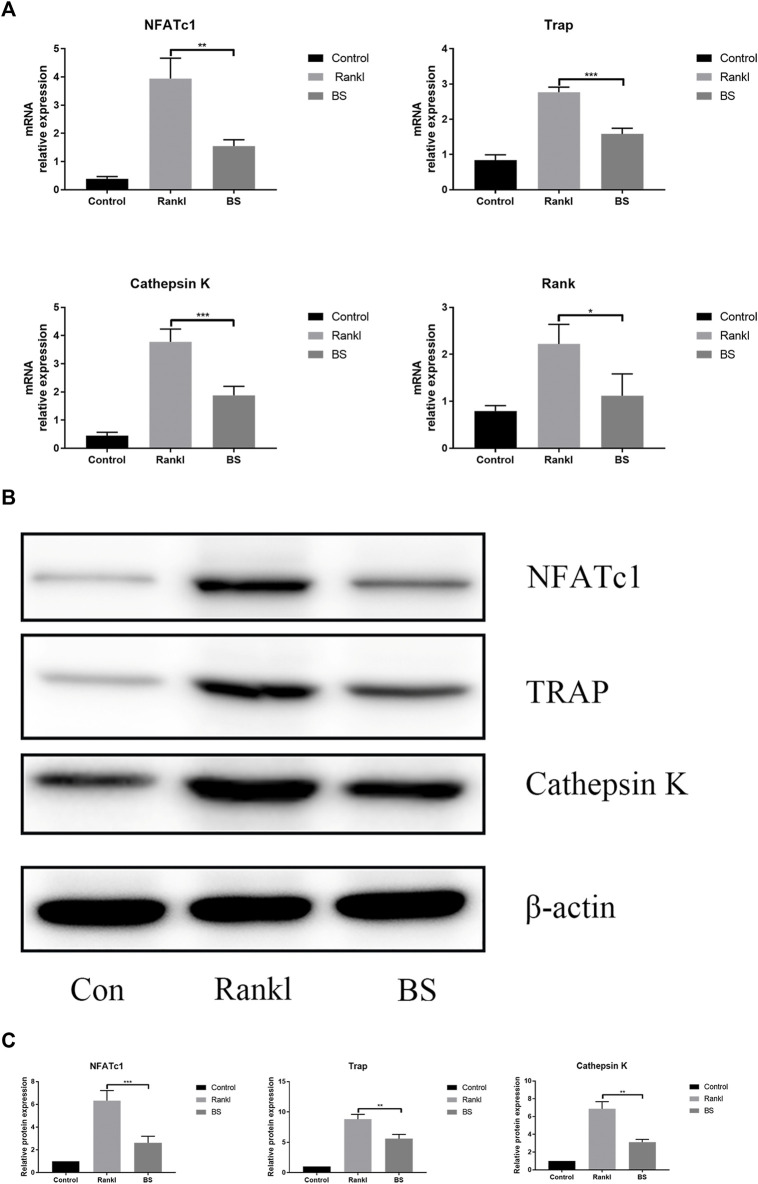
BS abrogates RANKL-associated NFATc1 activation and downregulates osteoclast-related genes. **(A)** NFATc1, TRAP, Rank, and Cathepsin K mRNA level. **(B)** Representative western blot of NFATc1, TRAP, and Cathepsin K in BS-treated BMMs. **(C)** Quantitative analysis of NFATc1, TRAP, and Cathepsin K. All bar graphs are presented as mean ± SD; n = 3. ∗*p* < 0.05; ∗∗*p* < 0.01; ∗∗∗*p* < 0.001.

After 3 days of stimulation, the mRNA expression levels of NFATc1 and osteoclast-related genes were measured, and the results showed that BS markedly suppressed the mRNA expression levels of NFATc1, TRAP, cathepsin K, and Rank (Figure 6A). On the fifth day of stimulation, the protein expression levels of NFATc1, TRAP, and cathepsin K induced by RANKL were suppressed by BS (Figures 6B,C).

### Baohuoside Ⅰ Attenuated Receptor Activator of Nuclear Factor-kB Ligand–Induced Urokinase-Type Plasminogen Activator Receptor Expression in Bone Marrow Monocytes

**FIGURE 7:**
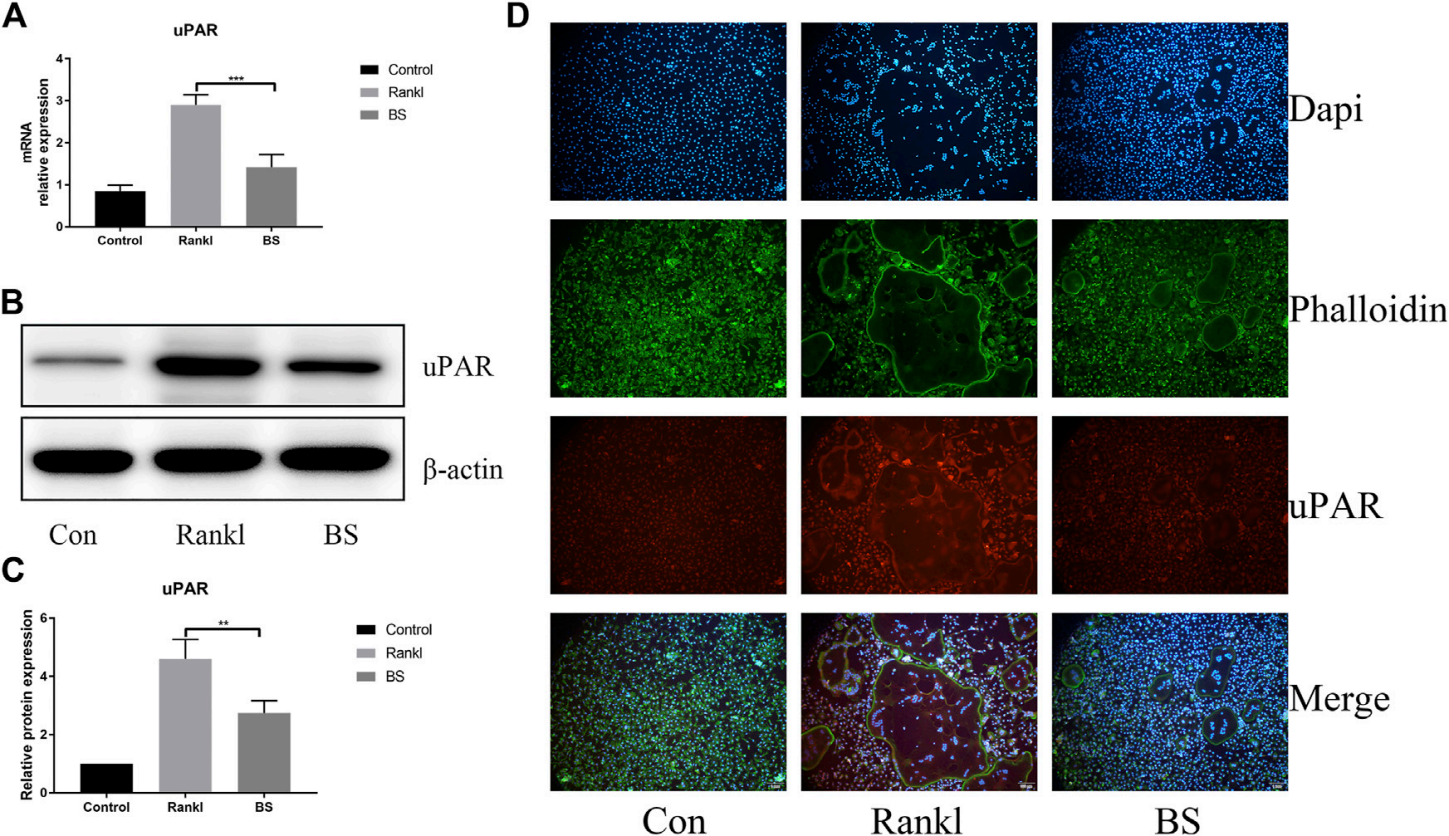
BS attenuated RANKL-induced uPAR expression in BMMs. **(A)** uPAR mRNA level. **(B)** Representative western blot of uPAR treated with BS **(C)** Quantitative analysis of uPAR. **(D)** Immunofluorescence images for effects of BS on the protein expression of uPAR. All bar graphs are presented as mean ± SD; n = 3. ∗∗*p* < 0.01; ∗∗∗*p* < 0.001.

Our previous study demonstrated the extremely important role of uPAR in osteoclast differentiation (Huang et al., 2018b). In this study, we investigated the effect of BS on the expression of uPAR during RANKL-induced osteoclast differentiation and found that BS treatment significantly downregulated the mRNA and protein expressions of uPAR (Figures 7A–C); immunofluorescence staining also demonstrated that the enhanced fluorescence intensity of uPAR induced by RANKL was decreased following the treatment with BS (Figure 7D).

### Baohuoside Ⅰ Inhibits Bone Loss, Osteoclast Activity, and Urokinase-Type Plasminogen Activator Receptor Expression Induced by Ovariectomy *In Vivo*

**FIGURE 8:**
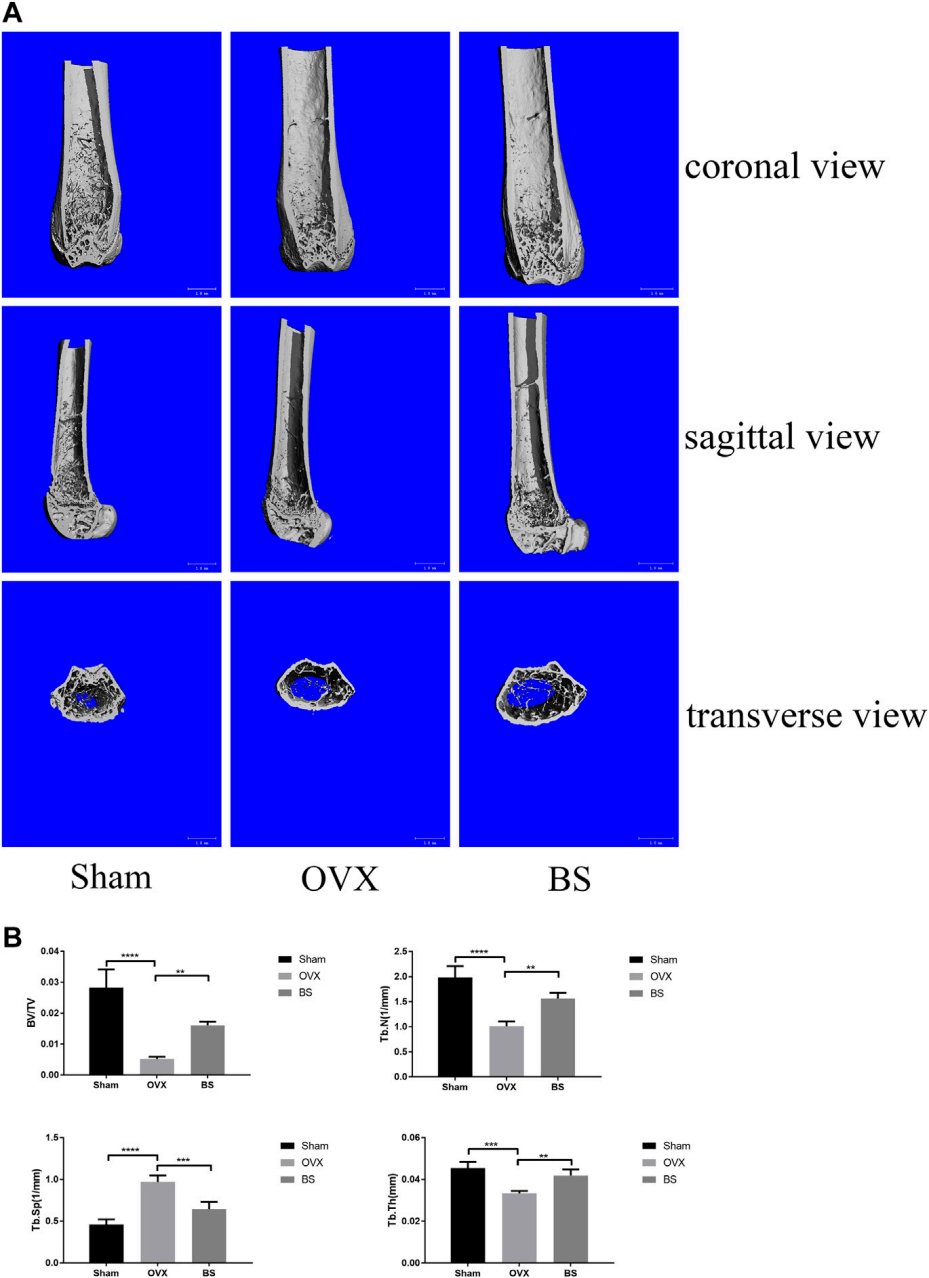
BS-inhibited ovariectomized–induced bone loss. **(A)** Representative 3D reconstruction micro-CT images of the trabecular bone of distal femoral metaphysis. **(B)** Quantitative micro-CT assessment of the trabecular structure: trabecular bone volume/tissue volume (BV/TV), trabecular number (Tb.N), trabecular thickness (Tb.Th), and trabecular separation (Tb.Sp). All bar graphs are presented as mean ± SD; n = 10. ∗∗*p* < 0.01; ∗∗∗*p* < 0.001; ****p < 0.0001.

**FIGURE 9:**
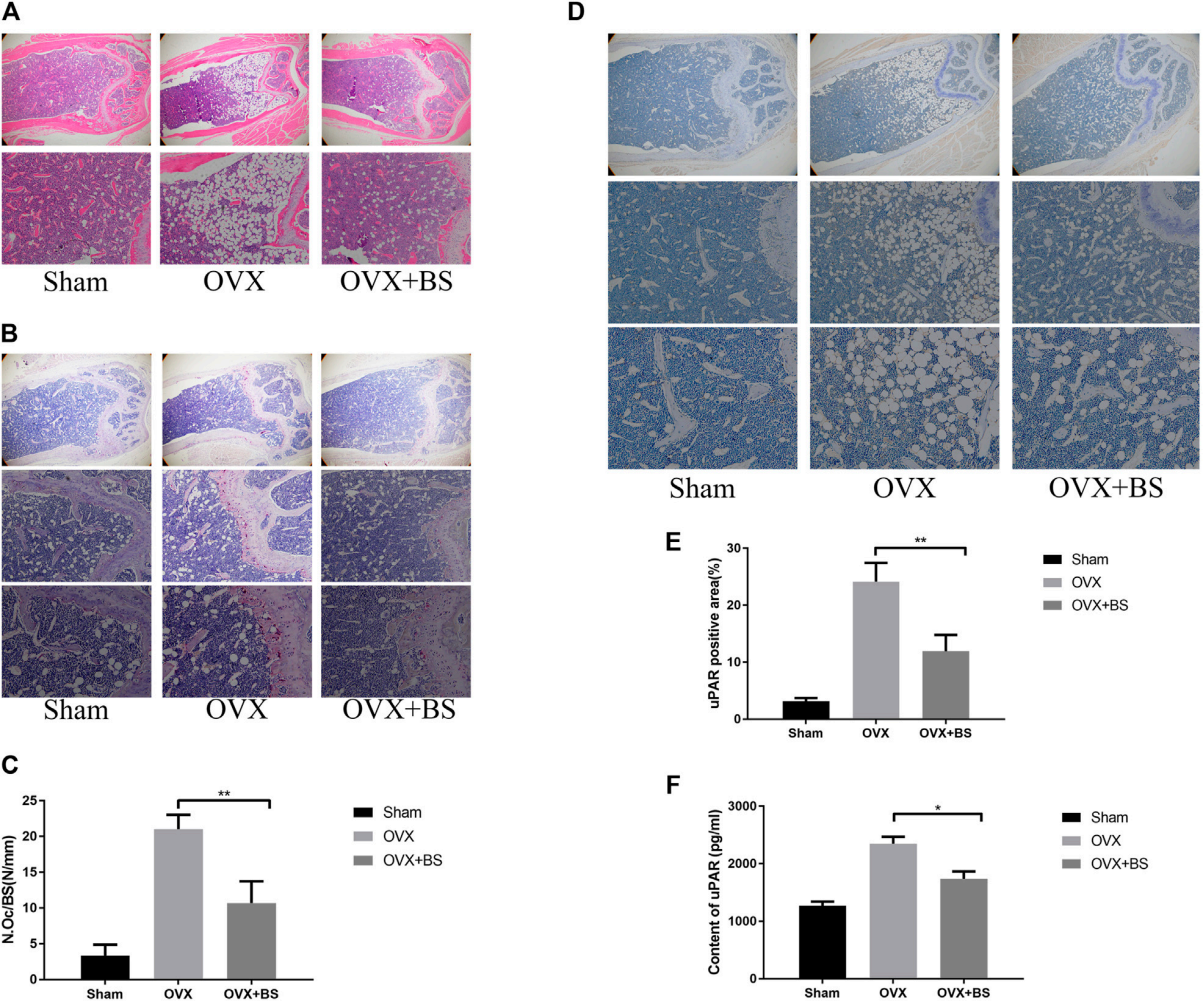
BS reduced osteoclast number and decreased the uPAR levels in OVX mice. **(A)** HE staining. **(B)** TRAP staining. **(C)** Quantitation of TRAP‐positive cells. **(D)** Immunohistochemical staining for uPAR. **(E)** Quantitation of uPAR‐positive cells. **(F)** The serum level of uPAR was detected by ELISA. All bar graphs are presented as mean ± SD; n = 10. ∗*p* < 0.05; ∗∗*p* < 0.01.

Based on the previous results of BS on the BMMs, we further assessed the actual effect of BS on preventing osteoporosis *in vivo*. Based on the three-dimensional (3D) reconstruction, the bone mass was significantly decreased in the ovariectomized mouse model, and the BS treatment could attenuate the bone loss caused by oestrogen deficiency (Figure 8A). Then, we analysed bone parameters after reconstruction and found that BV/TV, Tb.N, Tb.Th, and Tb.Sp decreased in the OVX group and increased after BS administration (Figure 8B). Consistently, H&E staining showed that BS treatment could prevent bone loss caused by OVX (Figure 9A). For osteoclast formation *in vivo*, TRAP Staining demonstrated that the quantity and number of osteoclasts per bone surface (N. Oc/BS) were increased in the OVX group and decreased in the BS group (Figures 9B,C). The *in vivo* expression of uPAR was elevated in the OVX group and dramatically reduced in the BS group (Figures 9D,E). Regarding the serum biomarkers, uPAR decreased in the BS group but increased in the OVX group (Figure 9F); bone resorption markers, namely, β-CTX and CTX-I, increased, and bone formation markers, namely, PINP, OPN, ON, and OCN, decreased after ovariectomy. BS downregulated β-CTX and CTX-I caused by OVX, resulting in bone loss, and upregulating ON and OCN, contributing to bone formation (Supplementary Figure S1).

## Discussion

Steadily accumulating evidence has demonstrated that icariin is a promising alternative therapy to prevent osteoporosis due to its regulatory role in bone formation and bone resorption (Ming et al., 2013; Huang et al., 2017). However, recent studies have reported that icariin has a very low oral bioavailability, and the material basis of icariin’s anti-osteoporosis effect is attributed to its metabolites of active components in the human body.

It has been reported that the bioavailability of 50 mg/kg icariin in rats *via* intragastric administration is less than 12%, and the peak concentration is only 162.4 μg/L (Xu et al., 2007). When orally administered, icariin should be hydrolysed into secondary glycosides in the small intestine prior to absorption. In a recent study, Zhou et al. (2013) reported that icariin is hydrolysed to icaritin *via* icariside I or baohuoside Ⅰ by the rat intestinal flora. In the current study, we initially investigated the efficacy of icariin and its main metabolites in inhibiting osteoclast differentiation. Consequently, we confirmed that icariin, baohuoside Ⅰ, icariside Ⅰ, and icaritin all inhibited osteoclast differentiation *in vitro*, but the inhibitory effects varied. During RANKL-induced osteoclast differentiation, icariin and its metabolites all inhibited osteoclast formation, and baohuoside I showed the best efficacy in inhibiting osteoclast differentiation at three different concentrations. As mentioned above, icariin can be transformed into several metabolites in the body. Previous studies have demonstrated that icariin can suppress bone loss induced by OVX, and we also observed that OVX-induced bone loss was alleviated by treatment with baohuoside I; thus, it is very possible that baohuoside I has a synergistic effect with the other metabolites of icariin on attenuating bone loss.

During osteoclast formation and maturation, the enhanced activity of the MAPK and NF-κB signalling pathways has been demonstrated to be an important regulatory factor. As shown in a previous study, the activation of NF-κB was responsible for the secretion of proinflammatory cytokines, which further promoted osteoclast differentiation (Zhou et al., 2006). In this study, the inhibitory effect of baohuoside I on osteoclastogenesis was confirmed by the inhibition of P65 and IκBα phosphorylation. MAPK is another important signalling pathway in osteoclast differentiation. Interrupting the phosphorylation of p38, JNK, and ERK with the corresponding antibodies or small-molecule antagonists demonstrates the exact inhibitory effect on osteoclast formation (Wang et al., 2018; Chen et al., 2019; Zhao et al., 2019). According to our results, RANKL-induced ERK, JNK, and p38 phosphorylation could be markedly suppressed by baohuoside I. The activated MAPK and NF-κB subsequently initiate the transcription of nuclear factor of the activated T-cell cytoplasmic 1 (NFATc1), which further stimulates the expression of genes responsible for osteoclast function. Our results revealed that baohuoside I reduced the expression of NFATc1 and influenced the expression of TRAP, cathepsin K, and RANK. These findings indicate that baohuoside I downregulates gene expression, contributing to osteoclast formation and function by modulating the activity of the upstream MAPK and NF-κB pathways, ultimately resulting in inhibited osteoclast differentiation.

uPAR is a 55–70 kDa glycoprotein commonly located in the cell membrane through glycosyl phosphatidylinositol anchors (Rao et al., 2013). uPAR was reported to be a potent regulator of cellular adhesion, differentiation, proliferation, migration, and cell survival (Smith and Marshall, 2010; Huang et al., 2018b). An increased level of uPAR was observed in the synovial fluid and serum from rheumatoid arthritis patients, and the fibroblast-like synoviocytes with an elevated uPAR showed more significant proliferation, migration, and invasiveness (Liu et al., 2018). In breast cancer, the administration of a uPAR antibody (huATN-658) significantly reduced primary tumour growth and skeletal lesions (Mahmood et al., 2020). In the bone tissue, our previous study confirmed that downregulating uPAR through siRNA could suppress osteoclast formation, and Kanno et al. (2016) reported that the master transcription factor NFATc1 was downstream of uPAR (Rao et al., 2013). In our present study, we observed that baohuoside I not only attenuated bone loss but also reduced the expression level of uPAR in the bone marrow. *In vitro*, RANKL stimulation induced uPAR expression, which is in line with previous reports, and baohuoside I inhibited osteoclast differentiation accompanied by reduced expression of uPAR. All these results indicated that the molecular mechanism by which baohuoside I inhibits osteoclasts lies in its modulatory effect on uPAR.

However, several important limitations still exist in our study. Our study clarified that baohuoside I showed the best efficacy in osteoclastogenesis inhibition, osteoclast marker regulation, and bone resorption suppression after the administration of baohuoside I *in vivo*. The balance between osteoclastogenesis and osteogenesis is responsible for fine-tuned skeletal remodelling. For bone formation, baohuoside I was proven to facilitate orientation osteogenic differentiation of the bone marrow mesenchymal stem cells (Luo et al., 2018). Moreover, the low serum bone formation marker in OVX mice was improved by treatment with baohuoside I; thus, it seems that baohuoside I has a similar effect to icariin on bone metabolism in that it may improve bone mass by stimulating bone formation and suppressing bone resorption (Kim et al., 2018; Xu et al., 2019; Yu et al., 2020). The mechanism of action of baohuoside I on osteogenesis requires further exploration. In addition, as a potential mediator, the exact role of uPAR in osteoclast differentiation needs to be further clarified.

In summary, our study demonstrated that baohuoside I protected against bone loss from ovariectomy and attenuated the expression of NFATc1 and uPAR induced by RANKL stimulation. These results suggested that baohuoside I may serve as a latent therapeutic strategy for osteoporosis.

## Data Availability

The original contributions presented in the study are included in the article/Supplementary Material, further inquiries can be directed to the corresponding authors.

## Supplementary Material

Supplementary Figure S1 BS reduced serum level of bone-resorption makers and partly enhanced level of serum bone-formation makers in OVX mice. The serum level of β-CTX(a), CTX-I(b), PINP(c), OPN(d), ON(e) and OCN(f) was detected by ELISA. All bar graphs are presented as mean ± SD; n = 10. ∗*p* < 0.05; ∗∗*p* < 0.01.
