# Supplementary figures and images for: Baohuoside I Inhibits Osteoclastogenesis and Protects Against Ovariectomy-Induced Bone Loss
Source: Front Pharmacol. 2022 Apr 27;13:874952. doi: 10.3389/fphar.2022.874952 (PMC9092047; doi:10.3389/fphar.2022.874952)

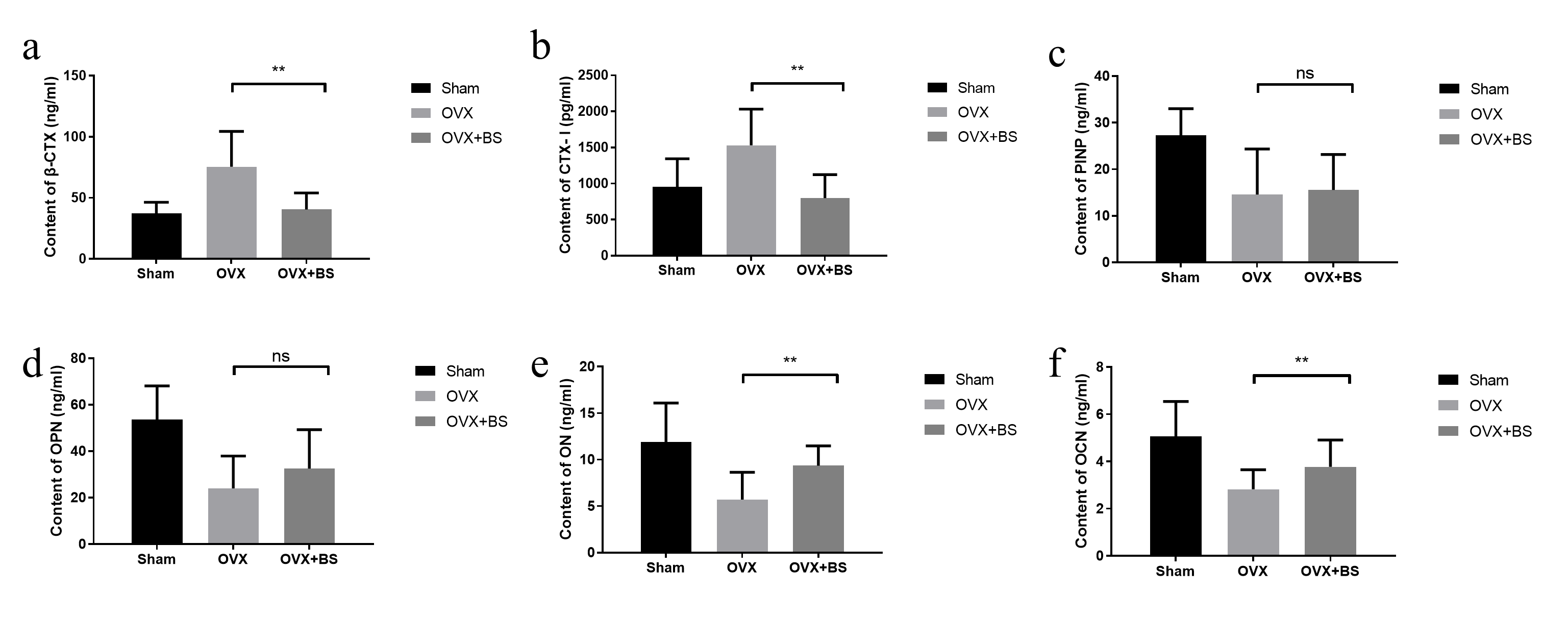

Supplement: Supplementary file 1 [file Image1.TIF]
